# The Role of Emotion Regulation as a Potential Mediator between Secondary Traumatic Stress, Burnout, and Compassion Satisfaction in Professionals Working in the Forced Migration Field

**DOI:** 10.3390/ijerph20032266

**Published:** 2023-01-27

**Authors:** Francesca Tessitore, Alessia Caffieri, Anna Parola, Mauro Cozzolino, Giorgia Margherita

**Affiliations:** 1 Department of Humanities, Philosophical and Educational Sciences, University of Salerno, 84084 Fisciano, Italy; 2 Department of Humanistic Studies, University of Naples Federico II, 80138 Naples, Italy

**Keywords:** secondary traumatic stress, emotional regulation, forced migration, mediation model, burnout, compassion satisfaction

## Abstract

Background: Professionals working with refugees are vicariously exposed to complex traumatic experiences lived by forced migrants, which can lead to the risk of developing secondary traumatic stress, burnout, and a poor compassion satisfaction. The current study aimed to explore the effects of secondary traumatic stress on burnout and compassion satisfaction in 264 Italian professionals working with refugees and forced migrants. Moreover, it examined the mediating role of emotion regulation between secondary traumatic stress and both burnout and compassion satisfaction. Methods: A structural equation modeling (SEM) was performed to test the hypothesis. Results: The results showed that increased levels of secondary traumatic stress were associated with an increase of burnout both directly and indirectly through the mediation of emotion regulation. Emotion regulation also totally mediated the relationship between secondary traumatic stress and compassion satisfaction. Conclusions: The results suggested that prevention or interventions programs targeting emotion regulation might be important to improve the professional quality of life of operators working with refugees and forced migrants.

## 1. Introduction

In line with the role covered in the past, Italy, due to its geographical position, continues to assume a central part in the migratory flows. Currently, the Italian reception system for forced migrants has a multilevel and complex structure, including “hotspots” (Italian acronym: CPA), settled near to the ports of landing and aimed at identifying the irregular migrants and managing their international protection requests as well as centers of first and second reception, the so-called Extraordinary Reception Centers (Italian acronym: CAS) and the Reception and Integration System (Italian acronym: SAI) that host the asylum seekers waiting for the evaluation of their asylum claim and the refugees who have already achieved the refugee status helping them during their inclusion path, respectively.

Several multidisciplinary professional figures work with forced migrants in these centers in Italy, as well as in others European countries [1]. Among them are health workers, lawyers, social operators, psychologists, educators, and cultural mediators. There are specific mandatory duties required by the different professions involved in providing assistance. However, all these professionals share intertwined social and ethical challenges. These challenges concern promoting social justice, defending human rights, and taking care of the forced migrants’ health, whilst trying to engage with human suffering deriving from the cumulative severe traumatic experiences lived by refugees and forced migrants [2,3,4,5]. Professionals in this field are not always well equipped to respond to the refugees’ needs [6], since their professional well-being continues to remain a marginalized area of investment due to the paucity and precariousness of resources destined for these services [7].

Social, organizational, group, and individual elements contribute to the complex functioning of migration services and to the experience lived by operators [8].

The professionals working with the traumatized population were reported to be at an increased risk of developing secondary traumatic stress [9,10]. Secondary traumatic stress (STS) is conceptualized as the reaction to the emotional demands on service providers from exposure to traumatic images, affect, and memories expressed by trauma survivors [11,12]. In the context of forced migration, tortures, rapes, enslavements, persecutions, as well as human or sexual trafficking, represent just few examples of the cumulative traumatic experiences lived by asylum seekers and refugees before, during, and sometimes after their migration journeys. In this sense, the operators working in this field, being constantly exposed to these lived experiences, often become the “first” able to see and recognize the traumatic value of the refugees’ experiences [2] and end up “incubating” the trauma in their minds [13]. The exposure to the multidimensional structure of “refugee trauma” [14] as well as this “digestive” function drives the high possibility for professionals to develop secondary traumatic stress symptoms [15,16,17].

Several studies investigating the mental health of professionals working in the field of migration have also shown a poor professional quality of life, with high levels of burnout, dissatisfaction, and fatigue [18,19,20]. The relationship between secondary traumatic stress and burnout is well known in literature [21], even though the direction of this relationship often depends on the context of application. Within the professional quality of life framework, burnout is defined as a mix of feelings of hopelessness and difficulties in dealing with job duties [22]. Researchers that investigated burnout among professionals working in the field of migration highlighted feelings of mistrust, inadequacy, and anger. Furthermore, the constant contact with the emotions of anger and sadness expressed by the refugees was identified as the main factor influencing the burnout levels of professionals [20,23].

Within the theoretical perspective developed by Stamm [22], the construct of the professional quality of life is composed by two sub-domains: compassion satisfaction and compassion fatigue. The compassion satisfaction (CS) is defined as the feeling of well-being, gratefulness, and growth originating from helping others [24,25]. Compassionate fatigue (CF) is interpreted as the negative slope of the professional quality of life of the individual. According to the Stamm [22] theorization, CF includes two sub-dimensions, burnout and secondary traumatic stress, which is defined, not as a disorder, but as a broad emotional vicarious response to trauma.

Currently, there is a large debate on the operationalization of these constructs. Some authors consider compassionate fatigue and secondary traumatic stress as constructs difficult to differentiate [11,12,20,26]. Cieslak et al. [21] found that when authors investigated the relationship between burnout and secondary traumatic stress within the compassion fatigue framework, a substantial overlap between these two measures emerged. Therefore, theoretically and methodologically, we decided to embrace the Cieslak et al. [21] perspective, considering secondary traumatic stress as being independent from the compassionate fatigue framework to better guarantee the distinction between measures and constructs.

The association between secondary traumatic stress, burnout, and compassion satisfaction was previously explored in the literature with different professionals working with traumatized patients or clients [21,27]. Măirean [27] hypothesized and showed that secondary traumatic stress was negatively associated with compassion satisfaction in Romanian health care workers. A meta-analysis by Cieslak et al. [21] showed the strong association between secondary traumatic stress and burnout, synthesizing data from different professionals working with trauma survivors (among them: healthcare workers, soldiers, therapists, social workers, and rescue workers). The authors of the meta-analysis also found that the relationship between secondary traumatic stress and burnout was predominantly stronger in female groups of participants. 

Moreover, the empathy-based stress process model [28] considered secondary traumatic stress as a predictor of burnout and other adverse occupational health and wellbeing outcomes. Research has widely shown the impact of secondary traumatic stress on burnout levels in nurses [29,30,31]. On the other hand, other studies highlighted how people working in nurseries or health care services, satisfied by their work, were less prone to develop secondary trauma symptoms [27,32,33,34].

Although the scientific literature showed a high-level of burnout and moderate-to-severe secondary traumatic stress in professionals working in the forced migration field [35], the relationship between these variables in the field of migration was poorly researched.

One study showed the association between secondary traumatic stress and burnout in North Korean refugee service providers [31], whereas another study showed that both high secondary traumatic stress and high compassion satisfaction could be found in professionals working with Mexican and Central American refugees [36]

The first aim of the present study was, therefore, to understand in-depth the relationship between secondary traumatic stress, burnout, and compassion satisfaction in the context of professionals working with forced migrants.

Additionally, as seen in studies on people with post-traumatic stress symptoms, trauma, especially that of an interpersonal nature, produces a disruption of the processes aimed at regulating the effect within individuals [37,38,39]. In different approaches, the adverse impact of relational trauma on the development of an emotion regulation system was demonstrated [40,41,42,43,44]. Emotion regulation is a multifaceted construct consisting of acceptance of emotional response, continued ability to engage in goal-directed behavior, impulse control, emotional awareness, utilization of regulation strategies to change or reduce the intensity of the emotion, and emotional clarity [45]. Two different hypotheses regarding the relationship between emotion regulation and trauma were described in the literature. First, a number of authors suggested that emotion regulation difficulties are one of the complex symptoms that specifically develop after interpersonal trauma [46,47]. Secondly, in general, post-traumatic stress symptoms have been suggested to be related to emotion regulation difficulties [48]. To date, emotion regulation was included among the main resilience factors in the domain of self-regulation in cases of secondary traumatic stress disorder [49]. To the best of our knowledge, no study has examined the relationship between secondary traumatic stress and emotion regulation in professionals working with refugees. Consequently, a second goal of the present study is to understand and evaluate the relationship between secondary traumatic stress and emotion regulation in professionals working in the migration field.

Although previous studies highlighted the socio-demographic and policy risk factors for the quality of life of professionals working with forced migrants, we were also interested in detecting the psychological factors that could promote a better professional quality of life of this population. In this sense, different studies showed the impact of maladaptive emotion regulation on burnout experienced by different professional populations such as foreign language teachers and healthcare workers [50,51,52,53]. Studies with mixed samples also showed how the suppression of emotion could be detrimental for the job satisfaction [54]. Instead, the impact of reappraisal emotional strategies was less studied on professional satisfaction, though it showed elsewhere its powerful effect on general life satisfaction [55]. To our knowledge, no prior study has assessed the association between emotion regulation, burnout, and compassion satisfaction in professionals working with migrants. Consequently, the original third goal of the current study will be to clarify the effect of emotion regulation on burnout and compassion satisfaction in professionals working in the migration field.

A hypothesis on the relationship between secondary traumatic stress, emotion regulation, and compassion satisfaction in healthcare workers could be found in Măirean’s [27] work; the study showed how the intrusion—considered part of secondary traumatic stress —was predicted by expressive suppression (a specific emotion regulation strategy based on the suppression of emotions) with the moderate effect of compassion satisfaction. Hence, the study highlighted that healthcare workers with a low tendency to use suppression and high compassion satisfaction were at lower risk of developing intrusions symptoms. No similar research aimed at creating and testing the hypothesis on the relationship between secondary traumatic stress, emotion regulation, burnout, and compassion satisfaction has been applied to professionals working with migrants.

Looking at the literature on trauma, the mediation effect of emotion regulation between past traumatic experiences and PTSD or other disorders has been widely shown [56,57,58]. Results on Syrian refugees also showed that good emotion regulation was mediated between the cumulative trauma experiences lived by forced migrants and the development of PTSD as well as the post-traumatic growth [59]. Focusing on operators, Benuto [50] showed that a good emotion regulation mediated the relationship between the secondary traumatic stress and the maladaptive coping strategies of protective service providers. Otherwise, interventions aimed at regulating the emotion regulation were effective in improving the mental health and social quality of life of vicariously traumatized people [60]. Hence, combining the previous results emerged from the literature, we hypothesized that emotion regulation could act as an important resilience factor mediating the relationship between secondary traumatic stress and burnout as well as the compassion satisfaction of professionals working with refugees and asylum seekers.

### Hypothesis

**Figure 1:**
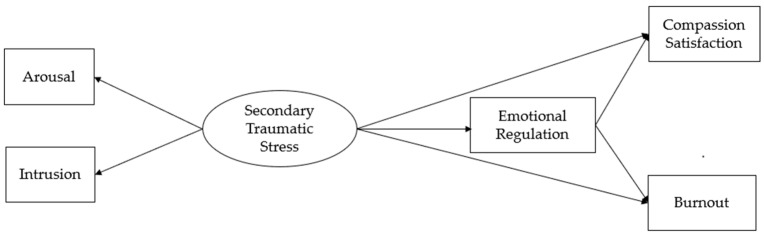
Hypothesized model.

First, we hypothesized that secondary traumatic stress significantly increases burnout and decreases the professional satisfaction of professionals working in the migration field. Second, we hypothesized that secondary traumatic stress, with its disrupting effect on the psychic elaboration processes, impairs the emotion regulation skills of professionals. We also hypothesized that difficulties in emotion regulation would be positively associated with professional burnout and negatively associated with professional satisfaction. Hence, we hypothesized that emotion regulation mediates the relationship between secondary traumatic stress and both burnout and compassion satisfaction in professionals that work with refugees and forced migrants (Figure 1).

## 2. Materials and Methods

### 2.1. Participants

Participants of the present study were male and female professionals working or collaborating with first and second reception centers (SAI and CAS centers) as well as with Third Sector Entities (non-profit organizations, social cooperatives, cultural associations) that offer services to forced migrants (single individuals as well as families) across the whole Italian territory. The survey was directed to employed, self-employed, and project workers, as well as volunteers who cover different professional roles (i.e., health workers, lawyers, social operators, psychologists, educators, and cultural mediators) within the considered institutions. We start from the idea that the operators, albeit with different roles, share the meanings of a socio-cultural system and, therefore, a homogeneous and unitary psychic functioning. A total of 264 workers participated in the research, 63 males (23.9%) and 201 females (76.1%) aged from 25 to 79 years (M = 39.39; SD = 9.59) (Table 1).

### 2.2. Measures

An ad hoc survey was developed for the purposes of the study. The survey was composed by a socio-demographic schedule, the scales measuring the compassion satisfaction and the burnout levels of the Italian Version of the Professional Quality of Life Scale (ProQOL) [22,61], the Italian version of the Secondary Stress Traumatic Scale (STSS) [62,63], and the Italian version of the Difficulties in Emotion Regulation Scale (DERS) [45,64] for a total of 73 items. The survey was developed and administered online via Google Modules.

The first part of the survey contained information about the study, its aims and procedures, the researchers contacts, and asked the participants to sign the informed consent. A second part collected some socio-demographic data along with some professional information. A third part included the self-report questionnaire as follows:

- The Professional Quality of Life Scale (ProQOL) [22,61]. The ProQOL is intended for use as a screening tool for the positive and negative aspects of working within a helping profession. It consists of three subscales: compassion satisfaction, secondary traumatic stress, and burnout, with the latter two subscales reflecting components of the construct of compassion fatigue. As mentioned above, in line with Cieslak et al. [21], in this study, the secondary traumatic stress was assumed as an independent construct from the compassion fatigue and was not measured with the ProQOL scale. For the purposes of the present study, therefore, we chose to administer only the scales measuring the compassion satisfaction (10 items) and the burnout (10 items) for a total of 20 items out of a total of 30 items. Participants were asked to respond to each item on a 5-point Likert scale from 1 (almost never) to 5 (almost always). Higher scores indicate higher levels of compassion satisfaction and burnout. In our sample, the Cronbach’s α were 0.877 for the compassion satisfaction scale 0.663 for burnout.
- The Secondary Traumatic Stress Scale (STSS) [62,63]. The Italian version of the STSS is a 15-item self-report that assesses frequency of intrusion (six items) and arousal (nine items) symptoms associated with secondary traumatic stress. Respondents were asked to report how frequently in the past 7 days they had experienced each item on a 5-point Likert scale from 1 (almost never) to 5 (almost always). Higher scores indicate greater levels of intrusion and arousal, and secondary traumatic stress. In our sample, the Cronbach’s α were 0.884 for the arousal, 0.771 for intrusion, and 0.903 for the total score of STSS.
- The Difficulties in Emotion Regulation Scale (DERS) [45,64] assesses individual differences in trait emotion dysregulation. The Italian version of the DERS consists of 36 items. For each item, participants were asked to indicate how often a particular statement applied to them on a 5-point Likert scale from 1 (almost never) to 5 (almost always). Scores on the DERS reflect levels of emotion dysregulation across six domains: (a) non-acceptance of emotional responses, (b) difficulties engaging in goal-directed behavior when distressed, (c) difficulties controlling impulsive behavior under negative emotional arousal, (d) poor emotional awareness, (e) limited access to effective emotion regulation strategies, (f) poor emotional clarity. Higher scores indicate greater levels of emotion dysregulation. In our sample, the Cronbach’s α was 0.942.

### 2.3. Data Analysis

As preliminary analyses, means and standard deviations were performed. Pearson correlation coefficient (r) was computed to evaluate the relationships between study variables. The internal consistencies were assessed by computing Cronbach’s α coefficients.

To test the model of relations, in which STSS predict CS and BO through the mediating effect of ER, structural equation modeling (SEM) was performed. STSS was set as a latent variable using Arousal and Intrusion as observed indicators. The dimensions of CS and BO were set as observed variables in line with the differences that the dimensions assume. Finally, as has already been done in previous studies [56,65,66,67], the ER was set as an observed variable and mediator in the model.

A normal distribution was found for all the study variables. Consequently, the maximum likelihood (ML) estimator was used to test the hypothesized SEM [68,69]. A 1000 bootstrap resampling procedure was applied. No missing data were found.

To evaluate the overall model fit, the comparative fit index (CFI) and the standardized root mean residual (SRMR) were used [68,69,70,71]. The following cut-off criteria were used to assess the goodness of fit: a CFI higher than 0.95 and an SRMR lower than 0.08 [68,70,71,72].

## 3. Results

Means, standard deviations, Cronbach’s α coefficients, and correlations are reported in Table 2. Cronbach’s α values confirmed the satisfactory internal consistency of each scale used. A correlation analysis showed that CS was negatively associated with burnout (*r* = −0.584), ER (*r* = −0.356), ARSL (*r* = −0.280), and the global dimension of STSS (*r* = −0.225); while BO was strongly positively associated with ER (*r* = 0.612), Arousal (*r* = 0.642), Intrusion (*r* = 0.464), and the global dimension of STSS (*r* = 0.627). The Arousal and Intrusion dimensions were strongly positively correlated with each other and with the overall STSS dimension.

**Figure 2:**
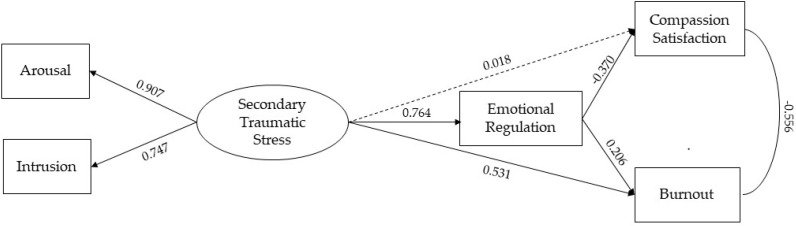
Graphical representation of the structural equation model.

The conceptual model was tested using SEM. Specifically, we tested whether the STSS, as a latent variable, is influenced directly and indirectly through the mediating effect of ER, CS, and BO. The hypothesized model (Figure 2, Table 3) provided adequate goodness-of-fit indices: CFI = 0.982; SRMR = 0.031. As shown in Figure 2, STSS predicted ER (β = 0.764; SE = 0.043; *p* < 0.001) and BO dimensions (β = 0.531; SE = 0.089; *p* < 0.001). Moreover, a significative direct effect of ER on CS (β = −0.370; SE = 0.112; *p* < 0.001) and BO (β = 0.206; SE = 0.092; *p* = 0.025) were found.

Bootstrapping analysis indicates that the indirect effects were significant: STSS to CS via ER (β = −0.282; SE = 0.080; *p* < 0.001; CI [−0.470, −0.111]); STSS to BO via ER (β = 0.158; SE = 0.068; *p* = 0.021 CI [0.009, 0.284]).

## 4. Discussion

The present study aimed to investigate the effect of secondary traumatic stress on burnout and compassion satisfaction through the mediation of emotion regulation in professionals working in the forced migration field.

Regarding the correlation analyses, our findings showed a strong positive association between the secondary traumatic stress, burnout, and emotion regulation, consistent with previous literature [50,73,74]. Coherently, in line with previous evidence [34,75], a negative week correlation emerged between the sub-dimension of avoidance of the secondary traumatic stress and compassion satisfaction. Surprisingly and in contrast with the previous literature [27], the results showed no significant association between the sub-dimension of intrusion of the secondary traumatic stress and compassion satisfaction.

Our first hypothesis was that secondary traumatic stress significantly increases burnout and decreases the professional satisfaction of professionals working in the migration field. This hypothesis was only partially confirmed. On the one hand, in line with previous studies carried out on other “helping” professionals [21,29,76], our results confirmed that higher levels of secondary traumatic stress increased the burnout levels in participants. In fact, professionals working with forced migrants are required to assist refugees and asylum seekers in several complex situations. The exposure to the forced migrants’ trauma and the paucity of system resources can constitute a hard challenge, which can increase burnout levels. On the other hand, a non-significant effect of secondary traumatic stress on compassion satisfaction was found. This result is surprising because previous literature showed an association between secondary traumatic stress and compassion satisfaction in rescuers, and trauma and pediatric nurses [32,33,77]. Different interpretations can be formulated on this point. Among them, an in-depth study conducted with operators working with Mexican and Central American refugees showed that the exposure to the migrants’ traumatic experiences increased both the risk of secondary traumatic stress as well as compassion satisfaction [36]. According to Lusk and Tarrazas [36], this result was explained by the fact that professionals working in the field of forced migration are not only exposed to the refugees’ trauma but also to the refugees’ resilience, in the face of the complex experiences of violence that forced migrants lives. In addition, the PROQL framework suggested that secondary traumatic stress can be found in association with a normal or high compassion satisfaction in professionals working in high-risk situations such as war and civil violence contexts [22]. Research with professionals working with migrants in Italy showed that operators embracing an idealized professional profile could be satisfied by their work as well as being unable to recognize physical and mental fatigue [78]. Moreover, in the field of forced migration, not only personal resources but also all relational and contextual variables such as the work climate, the surviving experience of resistance in intersubjective terms might assume an important role in preserving the professional quality of life of the operators [3,14,79]. In any case, the relationship between secondary traumatic stress and compassion satisfaction remains an interesting area which needs to be further explored.

We would like to highlight that, from our point of view, both the absence of significant associations between the sub-dimension of intrusion of the secondary traumatic stress and compassion satisfaction as well as the non-significant effect of secondary traumatic stress on compassion satisfaction support the hypothesis that trauma with its disorganizing effect does not determine a linear relationship between psychic processes and, therefore, between our investigated variables. In this sense, for example, the distress caused by vicarious trauma and feelings of altruism and meaningfulness for the job independently coexist, configuring a sort of “dissociative” process in which the emotional burden of trauma and the necessity to preserve the meaning of one’s work through caring for the other, travel on parallel tracks.

Furthermore, our second hypothesis was that secondary traumatic stress, with its disrupting effect on the psychic elaboration processes, impairs the emotion regulation skills of professionals. In line with the wide literature on the impact of trauma on the psyche [47,80], the findings confirmed our hypothesis, showing the effect of secondary traumatic stress in predicting emotion dysregulation.

We also hypothesized that difficulties in emotion regulation would be positively associated with the professional burnout and negatively associated with the professional satisfaction. This hypothesis was also confirmed, in line with evidence obtained on other populations, such as foreign language teachers as well as healthcare workers [51,52,53]

Finally, our last hypothesis regarded the potential mediator role played by emotion regulation in the relationship between secondary traumatic stress and both burnout and compassion satisfaction. This hypothesis was also confirmed. In line with Benuto et al. [50], who studied the role of this psychic dimension on protective service workers, our findings confirmed the central protective role played by emotion regulation in increasing the professional quality of life of professionals working with forced migrants. It also confirmed the role of emotion regulation as a main resilience factor for helping professionals, regardless of their specific functions in social systems [49].

In summary, this study offered an in-depth exploration of the personal resources of professionals working in the forced migration field, investigating the relationship between secondary traumatic stress, burnout, compassion satisfaction, and the significant mediation role of emotion regulation.

From the authors point of view, this result has several practical implications from a preventive and clinical intervention perspective. First, findings suggest that working on improving emotion regulation skills and processes of operators exposed to the refugee trauma could reduce the risk of developing burnout. Furthermore, the results suggested that implementing supervision interventions that focus on the professionals’ emotion regulation will assist in improving the professional satisfaction of operators as well as their well-being [3,81,82]. Moreover, for people which presented secondary traumatic stress, tailored interventions aimed at promoting reflexivity on emotion regulation aspects could be very useful to improve higher professional quality of life. Studies on the efficacy of interventions on this population of operators are needed to support the implications of our study.

Several limitations must be acknowledged for this study. The cross-sectional nature of the study does not allow to establish a causal relationship between the variables. Therefore, future longitudinal studies are needed to support a more specific set of conclusions for the relationships found. Moreover, all the measures used were self-reported and the responses may have been influenced by well-known biases, such as social desirability. To fix this limitation, researchers in the future could use a mixed approach based also on qualitative investigations to allow for a more in-depth study of the variables. Additionally, the sample presents a high prevalence of females, which did not allow for a comparison the model across genders through multi-group analysis. Finally, the study refers to a sample of professionals working within the field of forced migration and does not differentiate between the different groups of professionals. Further studies could investigate the variables and their relationships comparing specific groups of professionals working in this field. Further studies investigating other samples of professionals working in other high emotional impact workplaces are also needed to determine the results’ generalizability.

Furthermore, the current study underlines the new hypothesis on the peculiar relationship between secondary traumatic stress and compassion satisfaction in professionals working in the forced migration field. Moreover, it highlights the role of emotion regulation as an intrapsychic process that in combination with environmental, organizational, and interpersonal factors can influence the quality of professional life for people working in the migration field.

In conclusion, from our point of view, the results highlight that the research within traumatic contexts needs to take into consideration the disruptive effect of trauma and the impact that trauma has on the non-linearity of psychic processes and, therefore, on the investigated variables.

## Figures and Tables

**Table 1:** Characteristics of participants.

Participants (*n* = 264)	Frequency (%)
*Sex*	
Male	63 (23.9)
Female	201 (76.1)
Mean age (SD)	39.39 (9.59)
*Type of employment*	
Employed	231 (87.5)
Self-employed	33 (12.5)
*Professional role*	
Legal workers	25 (9.5)
Caseworkers	32 (12.1)
Coordinators	26 (9.8)
Cultural mediators	29 (11.0)
Educators	43 (16.3)
Health workers (i.e., psychologists, doctors)	28 (10.6)
Social workers	81 (30.7)

**Table 2:** Preliminary analysis: Cronbach’s α, means, standard deviation, and correlations.

		α	M	SD	1	2	3	4	5	6
1	Compassion Satisfaction	0.877	39.15	0.56	-					
2	Burnout	0.663	22.28	4.76	−0.584 *	-				
3	Emotional regulation	0.942	61.53	19.65	−0.356 *	0.612 *	-			
4	Arousal	0.884	19.76	6.54	−0.280 *	0.642 *	0.693 *	-		
5	Intrusion	0.771	10.79	3.62	−0.077	0.464 *	0.569 *	0.677 *	-	
6	STSS	0.903	30.55	9.38	−0.225 *	0.627 *	0.703 *	0.959 *	0.858 *	-

Note. α = Cronbach’s α; M = mean; SD = standard deviation; * *p* < 0.001.

**Table 3:** Mediation model: direct and indirect effects.

	β	SE	95% CI [L–U]
STSS → ER	0.764	0.045	[0.665, 0.839]
STSS → CS	0.018	0.126	[−0.231, 0.264]
STSS → BO	0.531	0.090	[0.369, 0.719]
ER → CS	−0.370	0.112	[−0.590, −0.148
ER → BO	0.206	0.092	[0.012, 0.373]
Indirect effect of STSS to CS via ER	−0.282	0.080	[−0.470, −0.111]
Indirect effect of STSS to BO via ER	0.158	0.068	[0.009, 0.284]

Note: β = standardized beta; 95%CI = 95% confidence intervals (lower and upper bound).

## Data Availability

The data that support the findings of this study are available from the corresponding author upon reasonable request.
